# Supplementary material for: The impact of deceased donor maintenance on delayed kidney allograft function: A machine learning analysis
Source: PLoS One. 2020 Feb 6;15(2):e0228597. doi: 10.1371/journal.pone.0228597 (PMC7004552; doi:10.1371/journal.pone.0228597)
Supplement: Attachment — Submitted filename: Response to Reviewers.docx [file pone.0228597.s002.docx]

Fortaleza, November 9^th^ 2019

Dear Editor,

We are resubmitting our manuscript entitled **“The impact of deceased donor maintenance on delayed kidney allograft function: a machine learning analysis"** addressing all the issues raised by the reviewers. We appreciate all the comments, which contributed to the improvement of our manuscript, and thank you for the opportunity to resubmit this revised version.

The authors declare that data supporting the findings of this study are available as supplementary file.

Yours sincerely,

Tainá Veras de Sandes-Freitas

[**taina.sandes@gmail.com**](mailto:taina.sandes@gmail.com)

**Reviewer #1**: Costa et al have used machine learning to assess the effects of donor management on DGF and compared that to predictive figures from a multivariate algorithm. The study is clearly written and has a fair sample size. The approach using ML is new and inventive. The evaluation of the exact mathematical analysis is not my expertise.

I have some reservations though on the design of the study.

| 1. The focus of the study is a bit unclear to me. Since the title and introduction are on machine learning, I dont know why first a predicitive model is used to show the higher than expected incidence of DGF. Even if DGF is in the expected incidence a correlation with donor factors could be found. | We appreciate your comment and apologize for not being clear.  We used the predictive model as the starting point of the study only to test our hypothesis that probably variables not involved in these models could explain the high incidence of DGF in our country.  The following statement was included in Methods session:  *“To better test the hypothesis that variables not included in traditional DGF predictive models could explain the high incidence in our country, we opted to calculate the expected incidence of DGF using the nomogram described by Irish et al [1] as a starting point of the study.”* |
| --- | --- |
| 2. Data are very specific to countries or situations in which the DGF rate is this exceptionally high (for DBD). The mean KIT is very high and I dont think any European center will accept a KIT >21h for 50% of the transplants. The high DGF rate is cause by the long KIT untill proven otherwise, and not by donor management. | We totally agree with you that CIT has an unequivocal contribution to DGF in our country.  We then analyzed DGF incidence in CIT<21h subgroup and found 48.2%.  Theses results let us to imagine that there are other factors affecting initial graft function in our scenario.  We included this new result and strengthened the importance of CIT on Discussion session.  Results:  *“The incidences of DGF were 48.2% and 57.8% in CIT<21h and CIT≥21h subgroups, respectively.”*  Discussion:  *“The high CIT was notable and its contribution to DGF is unequivocal. The large territorial extension, the allocation model predominantly based on HLA compatibility, and the absence of specific allocation policies for “marginal” donors contribute to the long CIT in our country [23]. However, it is noteworthy that almost half of KT with CIT <21h presented DGF.”* |
| 3. Therefore I would like to question how sure you are that you are actually relating to donor management issues, since these donors are brain dead and dying. Are the factors analyzed, e.g. the need for inotropics or urine output, a measure for the quality of donor mamangement or simply a refelection of the severity of the illness of the donor or the phase in the brain dead process the donor is in? | You are absolutely right.  We included a statement on discussion/limitation addressing this issue.  *“Since we could not follow variables over time and assess adherence to care bundles, it is possible that some clinical and hemodynamic data reflected the severity of the disease that led to brain death and not the patient care.”* |

**Reviewer #2**: General Comments

| The rise of donation after circulatory death – where delayed graft function (DGF) in kidney transplants is common yet long term results are not inferior to donation after brain death, the end point of DGF is now considered much less important. That said, if donor maintenance can prevent DGF then that should of course be explored. | We totally agree with you, mainly in our scenario of DBD KT with too high DGF incidence. |
| --- | --- |
| I am unsure why DGF is so high in Brazil. You suggest because of poor donor management? Is there evidence of this? | We agree with you that our results do not allow us to robustly conclude that poor donor management is the main cause for DGF incidence in our scenario.  However, analyzes showed that hemodynamic variables are independently associated with DGF, suggesting poor patient/donor clinical situation, which could be due to: more severe disease, poor intensive care management before become a potential donor; or poor donor management.  We rewrote some sentences to clarify this issue.  *“Since we could not follow variables over time and assess adherence to care bundles, it is possible that some clinical and hemodynamic data reflected the severity of the disease that led to brain death and not the patient care. Besides, it’s possible that some clinical and hemodynamic parameters reflected patient situation before they became a consented donor, and thus may not reflect donor maintenance, but general intensive care.”*  *“Despite these limitations, this study brings important information on the contribution of clinical and hemodynamic patient and donor maintenance in the occurrence of DGF in brain-dead donors.”*  *“In conclusion, DGF incidence in Brazil is significantly higher than that predicted by available models and poor donor maintenance might explain this scenario, suggesting the need to improve potential donor care in our scenario. Although we believe that poor donor management is mainly a reflection of the poor economic conditions of our country, some educational actions could be taken, focusing on early recognition of potential donors and training staff who care for brain death patients.”* |
| I am not sure your study does show, as you suggest it does, that poor donor maintenance impacts on DGF. You have shown that donors with certain physiological parameters are associated with DGF. But you have not shown that the cause of the poor physiological parameters is a result of poor donor maintenance. They just may be a sicker cohort. One with more hypoxic injury – as evidenced by being more likely to have had a cardiac arrest – probably from out of hospital cardiac arrest. So, I don’t accept your conclusion. To prove your conclusion, you would have to examine adherence to a donor care bundle, or match cohorts between hospitals that manage donors differently.  Expanding this point, I would consider the following donor parameters may just reflect a sicker patient cohort: donor age, BMI, final sCr, cerebrovascular death, urine output, MAP, > 1 or high dose inotrope. Perhaps only blood glucose might be something the intensivist may have been able to control better. | We agree with your comment.  We included this discussion in text (see previous answer) |
| You describe in your methods ‘donor maintenance parameters’ but the results for which can be taken from anytime ‘during hospitalization’. So the worst result may be before the patient became a consented donor and thus may not reflect donor maintenance, but general intensive care. | Thank you for your comment. The following sentence was added to Discussion:  *“Besides, it’s possible that some clinical and hemodynamic parameters reflected patient situation before they became a consented donor, and thus may not reflect donor maintenance, but general intensive care.”* |
| A shame the machine perfused kidney transplant cohort were not included. That would be interesting to see if machine perfusion can mitigate against poor donor maintenance. | Once machine perfusion is not yet a reality in Brazil, we decided to use a more representative population in this study.  But we agree with you and we included the following sentence to discussion:  *“A cohort including machine perfused grafts may be useful to explore if pumping might mitigate kidney damage secondary to poor donor maintenance.”* |
| I was unclear what you hope can be done with your machine learning Figure 1? Is it useful because it describes the problem? Could it become a prediction tool – helping a transplant surgeon decide if a kidney needed machine perfusion? | Exactly! In figure 1, the “importance” of each variable is described.  Now we are developing a predictive model to help staff to decide about pumping, immunosuppression, and to planning hospital-stay. |

**Reviewer #2:** Specific and Minor Comments

| • You should say in your introduction (and ideally abstract) all were donors after brain death. | This information was added to abstract and introduction |
| --- | --- |
| ‘Due to the observational and retrospective nature of the study, with data anonymously analyzed, [the] informed consent was not obtained.’ No need for [the]. | "The" was removed from this sentence, as suggested. |
| Donor Maintenance. ‘poor hemodynamic conditions to which donors were submitted:’. This reads like someone submitted the donor to these problems, whereas they just may reflect the way things are and not negatively on donor maintenance. Example – reversed cardiac arrest. Was that cardiac arrest reversed in ICU. Or cardiac arrest reversed in the community leading to hypoxic brain injury and brain death. The first could represent poor donor maintenance. The second is juts the ways things are. | The sentence was edited, as following:  *“Donor maintenance parameters data showed the poor clinical and hemodynamic conditions experienced by donors”* |
| Should be ‘The challenge of properly maintaining potential deceased donors seems not to be exclusive of our population.’ | Thank you for our carefully review.  We corrected its sentence. |
