# Supplementary material for: The impact of deceased donor maintenance on delayed kidney allograft function: A machine learning analysis
Source: PLoS One. 2020 Feb 6;15(2):e0228597. doi: 10.1371/journal.pone.0228597 (PMC7004552; doi:10.1371/journal.pone.0228597)
Supplement: Attachment — Submitted filename: Response to Reviewers.docx [file pone.0228597.s003.docx]

ACADEMIC EDITOR:

| It would be very important that authors address the comments of reviewer 1 (and reviewer 2) in more detail, as there seems to be interest in the topic and the MS.  However, reviewer 1 remains very critical about the explantations given so far.  Also, the conclusions should probably be downsized significantly and limitations more clearly recognised by authors. | Thank you for the opportunity to detail and clarify our results.  All corrections suggested by reviewer 2 were incorporated to the text.  To better address the reviewer 1 concerns, we performed an additional test with our data, using and sensitivity analysis (elastic net regression). In line with machine learning results, this regression model suggested that some variables reflecting donor/patient care may be important.  Methods:  *“As an additional sensitivity analysis, we performed Elastic Net regression, a regularization model that mix Lasso and Ridge regressions. While Lasso regression enhances the prediction accuracy and interpretability of the statistical model by variable selection and regularization, Ridge regression improves prediction by shrinking large regression coefficients to reduce overfitting. Elastic net regression also provides a more interpretable model when compared to black-box results of ML methods. For this analysis, numerical predictors were normalized by a Box and Cox transformation and after center and scale. Median were imputed for missing values. Categorical predictors were dummy encoded. We used an alpha of 0.1 and 10-fold cross-validation to search lambda. After an optimal search of a lambda, we perform an elastic net with R package glmnet. Those coefficients inferior or greater than zero are considered relevant predictors. Variables whose coefficient was zero were considered not important for DGF prediction.”*  Results:  *“The results of Elastic Net regression are illustrated in Fig 1. Variables in red were risk factors for DGF and variables in blue were associated with reduced risk of DGF. This model presented better predictive performance when compared to logistic regression (AUC 0.749).”*  Discussion:  *“To further explore the contribution of other than the traditional variables to DGF incidence using a more interpretable model, we performed a sensitivity analysis using elastic net regression. Again, beyond the traditional conditions associated to DGF occurrence, variables reflecting donor management were risk factors (serum Na+, blood glucose) or protective (high dose vasopressors and diuresis).”*  We also improved the sentence about machine learning results.  *“The three statistical methods of better performance in analyzes using ML techniques were: boosted DT using C5.0 algorithm (AUC 0.791), boosting NN (AUC 0.886), and SVM with polynomial kernel (AUC 0.784). Fig 2 illustrates ML results and predictive performance. Variable are presented using the feature importance graph, in which higher the value of the feature/variable, more important it was to predict DGF. The feature importance was normalized between 0 and 1 by dividing by the sum of all feature importance values. As demonstrated in Fig 2, urine output, mean arterial pressure, and the use of more than one vasoactive drug or high dose vasopressor were predictive of DGF in all models. Blood glucose was predictive of DGF in DT and SVM models.”*  Finally, as you suggested, we downsized our conclusions and make it clear that these results are a start for future (and more robust) studies on this important issue.  *“In conclusion, DGF incidence in Brazil is significantly higher than that predicted by available models. Although our data do not allow us to draw definitive conclusions, this study suggests that donor illness severity and hemodynamic instability might contribute to this scenario. Prospective studies are needed to robustly conclude how donor management impacts on DGF incidence.”*  *“We believe bringing this issue up is crucial in our setting. Scant donor care is probably a reflection of the poor economic conditions of our country. However, some educational actions could be taken, focusing on early recognition of potential donors and training staff who care for brain death patients. “* |
| --- | --- |

Reviewer #1:

| I thank the author for the comments and explanations. They added explainig phrases to the manuscript. Unfortunately, the explanations the authors have given are not providing new insights.  Moreover, it appears that the conclusions of the article are preliminary.  For example, the contribution of KIT was not clarified, except for the statement that <21h was still 50% DGF.  The contribution of donor ilness severity was only speculated on. No data were added to test the hypothesis that donor managment or treatment was indeed in a causative relation to DGF.  I think the manuscript has too many uncertainties and speculations to make it acceptable in this form. | We really appreciate your concern in ensuring that good quality information is published.  So, to better address your concerns, we performed an additional test with our data, using and sensitivity analysis (elastic net regression). In line with machine learning results, this regression model suggested that some variables reflecting donor/patient care may be important.  Methods:  *“As an additional sensitivity analysis, we performed Elastic Net regression, a regularization model that mix Lasso and Ridge regressions. While Lasso regression enhances the prediction accuracy and interpretability of the statistical model by variable selection and regularization, Ridge regression improves prediction by shrinking large regression coefficients to reduce overfitting. Elastic net regression also provides a more interpretable model when compared to black-box results of ML methods. For this analysis, numerical predictors were normalized by a Box and Cox transformation and after center and scale. Median were imputed for missing values. Categorical predictors were dummy encoded. We used an alpha of 0.1 and 10-fold cross-validation to search lambda. After an optimal search of a lambda, we perform an elastic net with R package glmnet. Those coefficients inferior or greater than zero are considered relevant predictors. Variables whose coefficient was zero were considered not important for DGF prediction.”*  Results:  *“The results of Elastic Net regression are illustrated in Fig 1. Variables in red were risk factors for DGF and variables in blue were associated with reduced risk of DGF. This model presented better predictive performance when compared to logistic regression (AUC 0.749).”*  Discussion:  *“To further explore the contribution of other than the traditional variables to DGF incidence using a more interpretable model, we performed a sensitivity analysis using elastic net regression. Again, beyond the traditional conditions associated to DGF occurrence, variables reflecting donor management were risk factors (serum Na+, blood glucose) or protective (high dose vasopressors and diuresis).”*  You are totally correct about the unequivocal impact of CIT on DGF occurrence, even on transplants with short ischemia time. Each additional hour matters. However, it is probably (and these data preliminary suggest) that variables other than CIT are also important. We edit the following sentence on Discussion:  *“The high CIT was notable and its contribution to DGF is unequivocal. All statistical analysis demonstrated that each additional hour matters, even in KT with CIT < 21h. The large territorial extension, the allocation model predominantly based on HLA compatibility, and the absence of specific allocation policies for “marginal” donors contribute to the long CIT in our country. However, it is noteworthy that almost half of KT with CIT <21h presented DGF, suggesting the contribution of other factors beyond the CIT.”*  We recognize that our methodology is not robust and our data is not conclusive. However, these preliminary data are important to bring this issue to light. Future studies will certainly be needed to definitively confirm or refute our hypothesis. So, we downsized our conclusions and make it clear that these results are a start for future (and more robust) studies on this important issue.  *“In conclusion, DGF incidence in Brazil is significantly higher than that predicted by available models. Although our data do not allow us to draw definitive conclusions, this study suggests that donor illness severity and hemodynamic instability might contribute to this scenario. Prospective studies are needed to robustly conclude how donor management impacts on DGF incidence.”*  *“We believe bringing this issue up is crucial in our setting. Scant donor care is probably a reflection of the poor economic conditions of our country. However, some educational actions could be taken, focusing on early recognition of potential donors and training staff who care for brain death patients. “* |
| --- | --- |

Reviewer #2: General Comments

| Very much improved paper. Congratulations. | We thank you for your crucial contributions. |
| --- | --- |
| Specific and Minor Comments In Statistical analysis, better: ‘Diabetic donors were also excluded, since this was a near-zero variance predictor (all patients who received grafts from diabetic donors developed DGF).’ | The sentence was corrected as suggested: “presented” was replaced by “developed” |
| In Results, better: ‘The mean time of DGF was 11.8 ± 15.0 days’ | The sentence was corrected as suggested: “on” was replaced by “of” |
| Conclusion perhaps could be: ‘Additionally, a cohort including machine-perfused grafts may be useful to explore if pumping might mitigate kidney damage secondary to poor clinical and hemodynamic status.’ This is probably enough given you didn’t prove causation to donor management even if suggestive. | The sentence was corrected as suggested: “poor donor maintenance” was replaced by “poor donor clinical and hemodynamic status” |
